# Ten simple rules for running and managing virtual internships

**DOI:** 10.1371/journal.pcbi.1008599

**Published:** 2021-02-18

**Authors:** Johannes Werner, Debora Jeske

**Affiliations:** 1 Department of Biological Oceanography, Leibniz Institute of Baltic Sea Research, Rostock-Warnemünde, Germany; 2 High Performance and Cloud Computing Group, Zentrum für Datenverarbeitung (ZDV), Eberhard Karls University of Tübingen, Tübingen, Germany; 3 School of Applied Psychology, University College Cork, Cork, Republic of Ireland; Dassault Systemes BIOVIA, UNITED STATES

## Introduction

The importance of managing projects virtually and effectively has increased over time. Today, many research groups in the computational and natural sciences have become more international. In addition, many students are participating in virtual internships and research projects. This trend also extends to how students are supervised on various projects—often remotely and by faculty members or organizational supervisors outside their own institutions. In the following paper, the authors outline ten rules for any faculty interested in successfully running virtual projects with students from other institutions than their own. These recommendations build on existing guidance related to preparing for and hosting traditional on-site internships [1–3].

Virtual internships are computer-mediated internships where the intern works for an organization, usually employers, remotely. This means that these virtual interns and the organization are often located in different cities, countries, and even time zones. Many interns complete such internships to gain work experience or complete projects for academic credit [4]. Due to the ease of locating suitable talent for research projects, such internships are also of interest to many academics who seek suitable and motivated talent for research projects. Faculty members can play a similar supervisory role as the managers in organizations.

In the following section, we outline ten rules that can guide research supervisors in higher education and research institutions who are interested in running such virtual internships. These rules are relevant to both virtual and traditional internships that involve a dispersed team collaborating on a shared project. Nonetheless, the nature of virtual internships implies that project success is contingent on some aspects that are usually easier to track in traditional internships. Open communication, progress monitoring, proactive tool adoption, learning, and documentation are particularly critical in virtual settings to create transparency and facilitate project success. While some of these rules were created based on previous work we reference, none of the previous virtual internships focused on internships in research facilities. We believe that these rules can be readily applied to research projects focusing on computational biological analysis in multiple research disciplines as today’s technical infrastructure now allows labs and researchers to collaborate virtually on joint projects, using shared online resources and analytical tools.

In order to highlight the relevance of our rules, we included feedback and observations from three female and two male students who completed virtual projects in bioinformatics. Virtual internships are known in various fields; however, to our knowledge, this is the first time that virtual internships have been applied to the field of bioinformatics. In contrast to traditional wet lab internships, virtual internships in this research area can be offered if the supervisors provide the computational resources. All five interns completed their projects over the course of a three to six months’ period (with a time commitment of around 300 hours to 800 hours). Most virtual interns lived in Germany like their internship supervisor; however, they came from different educational institutions and lived in various locations.

### Rule 1: Preparation is everything

Project-based virtual internships require a significant amount of preparation to be successful. As a first step, supervisors need to identify suitable projects that can be completed in the time frame of an internship (several weeks to months). Moreover, supervisors have to create a road map that candidates can follow. In addition, supervisors need to consider the amount of time they can dedicate to training, meetings, and mentoring during the anticipated project time frame.

Supervisors need to be clear on the required candidate and technical skills as well as on the project time frame. In addition, it is important to clarify the technical specifications (hardware and software) as most interns will be using their own equipment rather than university resources to complete the projects. If the appropriate software is not available to the student via their own university network, temporary licenses will need to be made available by the supervisor to ensure that the successful candidates can complete the project in question (unless open-source solutions can be used instead).

A key aspect in the recruitment stage pertains to the identification of suitable target groups from which to engage in the selection process. If the project and thus training time is shorter, more skilled candidates may be preferred. Many students will be new to these internships, which means the supervisor will also have to introduce both project and virtual internships in general [5,6]. As soon as candidates have been selected, appropriate documentation is necessary to record the agreement between supervisor and student (e.g., project and task description, requirements around academic credit, potential remuneration, data management, project confidentiality, training or software agreements, and expected hours and time frame for the internship). This documentation may also be complemented by a learning agreement or a learning contract (since it is an internship after all), where mutual expectations around communication and supervisory support are outlined (e.g., mentoring).

### Rule 2: Clarify project and supervision characteristics

Clarifying what kind of project supervision and resources a supervisor can provide is helpful in setting expectations on both sides. It is quite easy for students to drop out of virtual internships (especially when they are not done for academic credit). Furthermore, past work [7] has shown that limited personal involvement and interaction with supervisors (including lack of feedback), passive supervision (where the supervisor always waits for the intern to initiate contact), one-sided communication, and no room for flexibility or skill development lead to less engagement on behalf of the intern. Due to the nature of virtual internships, involving geographic separation and computer-mediated communications, expectations may become harder to pin down when students come from different institutions, speak various languages, and bring very distinct experiences to the table. When supervisors keep these aspects in mind, they are more likely to achieve project success, and interns can have a more positive experience.

To ensure expectations are clear on both sides, it is imperative for supervisors and students to negotiate and agree the following:

1. weekly meeting frequency and commitment to meetings from both sides;
2. reporting and feedback frequency (how often the student submits work as well as time frame for feedback from the supervisor);
3. communication channels;
4. documentation requirements (e.g., coding, programming, problem-solving, and sharing of information over different platforms);
5. agreement on working periods, availability, and deadlines (in case of semester breaks, exam times, and holidays);
6. expectations around training participation and training engagement; and
7. plans for publication (if applicable) and other factors deemed relevant for the project.

### Rule 3: Be clear in your communication

An important step for a virtual project supervisor will be to make the implicit explicit. Open communication is critical in virtual internships to ensure that the dispersed team exchanges information effectively with one another while they are working on a project from different locations (and possibly even different time zones). Compared to traditional on-site projects, students completing a virtual internship may experience uncertainty and isolation when interactions are limited, and students might find it difficult to obtain feedback [7]. As a result, mistakes and confusion may arise when issues are not brought out into the open. This also applies to assumptions that supervisors and students hold about their projects, communication, and mutual expectations. Relevant evidence in support of this comes from the virtual internship literature. Virtual interns have often self-reported lower performance when they lack goal clarity and supervisory support. In addition, support satisfaction correlates positively with goal clarity and interns feeling valued [4].

The use of publicly and freely available tools can facilitate frequent exchanges, helping students to get assistance by checking in, which facilitates problem-solving when their projects hit a snag. Background information, task management, repository, and code review can be handled on GitHub or GitLab. Communication tools (e.g., Slack or Mattermost) facilitate the discussion within the team, whereas group meetings or longer discussion can also be scheduled via video conferencing tools such as Zoom, Microsoft Teams, or Jitsi. Project and communication needs determine which tools are most suitable.

In order to support communication, a two-way feedback system was implemented. Students received progress-related feedback and they were, in turn, encouraged to provide feedback to the supervisor via various tools (see “Rule 4: Identify and monitor progress on learning goals”). This also helped the supervisor gain more experience as these internships were a pilot project. To our knowledge, virtual internships in bioinformatics are a novelty. Regular meetings throughout the internship are also key to discuss progress, expectations, feedback, and address potential problems as part of the learning process.

#### Students’ observations

The importance of addressing assumptions and uncertainty was also noted by our students. Accordingly, one student noted that “check-ups over the phone or video conferences are best when complex issues need to be understood.” Another student noted that communication can create more clarity about project features, especially at the beginning: “The look over intermediate results and checkup, whether everything was understood, is important in the beginning to pave the way for individual work and new ideas.”

### Rule 4: Identify and monitor progress on learning goals

Students looking for virtual internships will seek these experiences for two main reasons: academic credit and work experience. Being clear on the project aims but also the learning goals will be critical to ensure that the internships are indeed the learning experiences that they are intended to be. In virtual internships, project progress and learning often takes place on an individual basis, making it potentially invisible to others unless efforts are made to highlight this. According to Rule 1, having a learning agreement in place ensures that expectations are captured and mutually agreed, creating specific learning goals to guide the training and skill development over the coming weeks and months. These goals usually specify what the student expects to learn, the training and support that supervisors will provide, as well as the options of mentoring and introductions to further career development opportunities.

Selection of highly skilled students can reduce the training requirements and burden for the supervisor. However, finding the perfect candidate is a difficult task. It is also worthwhile encouraging interns to be proactive about creating their own learning resources. Thankfully, several useful resources are already available to guide supervisors and students [8–11]. Intermediate objectives and a final deadline can further be helpful for motivation purposes. A clear structure and frequent reporting ensure that the supervisor can provide feedback as progress is being made, helping students to learn as they are achieving the various goals—rather than leaving feedback to the end when the project has progressed significantly or perhaps even ended. Naturally, the importance is that performance monitoring serves feedback and developmental purposes, rather than to exercise control over students. Feedback and reporting rounds support continuous learning and further contribute to the successful achievement of each project stage, ensuring completion of the project.

#### Students’ observations

Clear goals, support, and structure can help students to maintain their motivation over time and work effectively. While our students emphasized having a distraction-free work environment, many also found that setting a schedule helped them to stay on point: “Weekly reports and individual project check-ups were definitely motivating and beneficial for the work at home, especially when the amount of checks/requests are neither too rare nor too often.” Another student noted: “I would advise to set a schedule with goals for the time frame of choice. […] it is easy to lose track of time. Therefore, establishing routines and a schedule can be very helpful to put pressure regarding time on oneself. Similarly, this strategy prevents one from getting distracted due to the absence of work colleagues in one’s surroundings because in order to achieve daily goals, one has to adhere to the established time limit.”

### Rule 5: Keep track of and celebrate learning moments from start to finish

Learning moments represent all those instances where a student makes an important connection or leap that enables them to tackle a challenge. In addition, learning moments can represent all those times where the student achieves a learning or project goal. Encouraging students to keep track of their learning moments over the course of their project-based virtual internship (from start to finish) serves multiple purposes: First, it raises their awareness of what they have accomplished themselves and which skills they utilized, build on, or have newly acquired. This is particularly relevant to virtual settings where learning moments may not be visible or noticeable to the team. Second, it creates a useful record for both the student and supervisor to reflect upon to assess learning and improvements—as well as potentially new learning goals for the next stage of a project. High-quality documentation may also be an excellent means to share insights in larger project groups (and set the stage for reproducibility of studies, successive improvement of training guides, and longer-term knowledge management over several project iterations). Third, it creates an excellent repository of potential anecdotes and examples that a student may later be able to share in interviews when they are asked to demonstrate how they have tackled a problem in the past. Finally, it can become a helpful memorandum for the supervisors when they write references or provide recommendations for certificates to the students during or after the project (see also “Rule 10: Evaluate and improve your projects and supervision over time”).

Documenting learning moments and the achievement of milestones also serve other purposes. This process helps students to see the connection between completing a virtual internship and their own personal learning journey. By articulating how completion of tasks provided avenues for learning and skill development, students often realize that they have engaged in learning by doing, even if that meant completing certain tasks they were struggling with or learning certain routines, programs, etc.

Such success may then be celebrated over the various online channels used for communication, through presentation of the work to other students or research groups, poster submissions to conferences, and similar. For supervisors, such learning moments generate excellent opportunities to assess their choice of students and the effectiveness of their supervision and training materials. Supervisors may also be able to better plan by monitoring the learning progress and even potentially identify avenues to motivate high achievers or look toward alternative ways to get those on track who are struggling. Especially, faculty in research-intensive institutions may find that by tracking the progress of their students on such projects, they can prescreen candidates for potential research appointments.

### Rule 6: Provide and encourage tool adoption and documentation

Most faculty will have access to several tools, repositories, and video conference systems to support tool adoption and project documentation. Choosing the right tools for each project and providing access are essential. In addition, supervisors will have to take a technical check at the beginning to ensure compatibility of software and identify the most suitable communication channel, schedule, and approach for a diverse group of students—for individual as well as group communication. Some experience with distance teaching and system compatibility will be helpful, particularly when students are part of different educational systems as this may have technical ramifications.

In our pilot study, interns had to learn how to write scientific documentation in English, to do a reasonable literature review, to use the Unix command line, version control, and write shell scripts, to code mostly in Python and R, to apply further bioinformatics tools and where these might be useful, to utilize cloud/cluster computation systems, and to identifying the technical capabilities of DevOps systems (i.e., GitLab) with continuous integration. If project time frames allow for longer training periods and your intern cohorts do not have substantial programming skills, several resources are publicly available where interns can learn coding skills (e.g., Codeacademy or freeCodeCamp).

#### Students’ observations

Many students relied prominently on GitHub and GitLab for their code repository and version control. For communication, the student group used on Slack and Mattermost which both allow an integration with GitLab. The use of both tools enabled students to consult each other and contact their supervisor in case of questions. In consequence, the students were given the experience of a team environment where they could also see each other’s questions and answers while still working on their own project. Depending on the licenses available to the students via their institutions, the supervisor in the current pilot study used different tools. These included Jitsi, Skype, Microsoft Teams, and Zoom. Other online resources that were heavily used by the students included online dictionaries, their computational cloud infrastructure (de.NBI cloud), and StackOverflow.

One of the students summarized his experience with these tools as follows: “The programs Mattermost and GitLab were essential for both communication and storage of information regarding the investigation. […] Mattermost provided an excellent method to reach out for questions and exchange information in groups as well as privately for organizational or individual matters. The focus of GitLab, on the other hand, lay mainly on the research itself. It laid out the structure for the whole analysis through the medium of issues, dividing the stages of the investigation; consequently, facilitating the posting and discussion of results or procedures. In addition, when it came to writing code, GitLab enabled me to document changes over time to keep track of the alterations of the code. This allows the programmer to try out alternative approaches with the possibility of merging new code or go back to an older version of the script.”

### Rule 7: Connect students with one another and across projects

Communication channels and group chats can help students to feel connected to each other and provide them with another channel of support. That this indeed matters to the students’ experience is demonstrated in the existing research as well. As virtual internships are not, by default, limiting faculty to supervising local students, research groups may feature students from various national and international institutions. In consequence, networking across labs, disciplines, and national boundaries becomes a real possibility—paving the way for international collaborations, mentoring, knowledge exchange, and other opportunities that may enrich the students’ experience during their internships.

Peer coaching is also a useful tool employed in larger virtual internship groups as it allows peers to learn from one another [12]. This suggests that shared communication channels in combination with mentoring can improve the learning and networking experience of students -and potentially their supervisors—while they are completing virtual projects as well. Teams may also add structure and prevent silent dropout, a known problem with virtual internships. Where students are working together, they will also have to effectively plan common goals and how they tackle certain tasks, especially when students’ projects are interdependent. For example, some concerns may arise when students share output with one another. In general, sharing of code and documentation is crucial; however, issues about copyright, intellectual property, and nondisclosure need to be addressed as well, depending on project and funding agency.

#### Students’ observations

The need to collaborate across projects was also seen positively by our bioinformatics students. For example, one reported the following: “What also helped me was the collaboration/exchange of information and help with another student who worked on a similar project.” Accordingly, it is important to inform interns about plagiarism rules, data security, and appropriate data management [13]. Concerns about code and documentation sharing are more likely to arise in team projects where individual team members are working on their own dissertations. As noted by one student, “when students have the chance to have insight into the intermediate results, such as codes, thesis, or reports, there might be the risk of copying some ideas/concepts without permission. Therefore, some issues/reports or things such as the thesis should be shared only with the supervisor.” In the current pilot project with the bioinformatics students, these concerns were discussed as part of the feedback rounds with all team members.

### Rule 8: Encourage proactivity and independent problem-solving

While research has shown that two out of three virtual interns work in teams [4], virtual projects like internships require students to manage uncertain situations effectively. Rather than relying on supervisors to notice when students are struggling with a specific task, virtual internships require students to take the initiative—as the supervisor may not be immediately available when a problem comes up. Providing the right infrastructure and resources is one option here. However, it is also important for students to become proactive and independent problem-solvers.

Supervisors taking the time to properly onboard interns is important to help interns get started. Furthermore, helping interns to familiarize themselves with the processes and software tools, and encouraging them to make attempts to solve problems independently where possible, will foster learning and proactivity. It will be a learning curve—but a great opportunity for students to get ready for future employment where such proactivity, time management, strategic planning, and commitment to continuous learning are all essential to success.

#### Students’ observations

A few bioinformatics students commented on the need to be proactive as well as independent. The following demonstrates this further: “The motivation, basic self-organization and the courage to ask things are very important. […] The student might work alone and not have the courage to question things or think out of the box.” Several students commented on the need to be able to make plans (and revise them if needed), to seek clarification on tasks while also taking the initiative—exhibiting a sense of ownership of the project in the process. Indeed, all students appreciated the degree of independence that was encouraged and provided as it gave them the ability to work on projects in their own time and according to their own schedule—an advantage of virtual projects over traditional and on-site research internships. This reiterates the importance of having realistic previews early on and expectation setting at the beginning to select only those candidates who will accept these requirements and thrive while working on a project in a virtual internship.

### Rule 9: Offer mentoring and guidance

Mentoring and guidance are important to success in virtual as well as traditional internships. Past work suggests that having access to mentors and experts can be considered an incentive for many virtual interns [12,14]. Many students know that mentors and experts may also serve as important role models for later career decision-making as they offer potential support for one’s career aspirations and access to information [15]. The research evidence also suggests that mentored interns increased their communication and strategic problem-solving skills. Mentored interns report more opportunity to share information and cooperate as well as receive help from others [16]. Specific guidance around setting up the appropriate documentation to guide the mentor–mentee relationship is already available [17].

Faculty supervising project students can offer mentoring and guidance in numerous ways. One is to set up mentoring provisions for all interns at the start of the project. Another option that faculty are often very familiar with is to use their own expertise to introduce students to potential career platforms, early career networks, student groups, and role models who may serve as a mentor for the course of the project, and even beyond. Many educational institutions also have career services and large alumni networks that may further enable interns to reach out to experts in their aspiring area of professionalization [18]. The more specialized the project and envisioned career track, the more appreciated are introductions to specialists in the field [19].

#### Students’ observations

A sample quote demonstrates how mentoring was supported via different communication channels such as Mattermost: “Since both mentor and mentee are able to exchange exact commands, results, code snippets, and also have access to all of these as long as they’re stored in any of the previously mentioned applications; the latter being, in my opinion, the main advantage over oral communication due to the possibility of recurrent review of whatever information was shared between the participating parties. Therefore, the mentor has all the necessary means to answer questions at any time, keep track of the mentee’s progress, give instructions, and comment at any stage of the investigation.”

### Rule 10: Evaluate and improve your projects and supervision over time

While both traditional and virtual internships are frequently viewed as benefiting students more than the organizations, the reality is that the supervision of virtual interns represents learning experiences as well as growth opportunities for faculty. Faculty get to support students from various faculties and institutions, potentially even students studying in different countries. Furthermore, via virtual internships, faculty now have opportunities to mentor talent from different disciplines. This can also set the stage for mutual learning, as faculty can learn from their mentees as well, as they can thus access new information about new processes, programs, and approaches that are utilized in different laboratories. Virtual interns often become unofficial ambassadors for the organizations they have worked for when they share their experience with their home institutions. This may then create new connections between faculty across different institutions, with the students serving as a bridge.

Many relationships between interns and supervisors do not end with the termination of the project. Once faculty start working with students from different institutions, this may pave the way to additional future collaborations and lasting connections (via joint papers or social networks). Students may also provide important feedback on the perceived quality and comprehensiveness of supervisory training materials, help to improve and adapt existing software guidance for specialized analyses, and may help supervisors to review and improve their general supervisory processes. In our case, the two-way feedback system and a feedback round at the end of the projects provided us with important insights for future virtual projects in bioinformatics.

Such student feedback can help faculty supervisors to revise and optimize their approaches for different projects and students (see also “Rule 5: Keep track of and celebrate learning moments from start to finish”). And as employers, students, and educators are increasingly becoming accustomed to online education and remote working modes, virtual internships are a good way to access talent across different countries before potentially recruiting them into research positions on-site. This has implications for future collaborations as past work has shown that 90% of interns who successfully completed virtual internships expressed an interest in taking on future virtual internship or career options [4]. This indicates that virtual internships can support the recruitment of talent, as well as help institutions—regardless of location and size—to increase linguistic, ethnic, and gender diversity while facilitating more complex team-based project work across multiple sites.

## Conclusion

The outlined “Ten simple rules” provide an overview of the aspects that are relevant to faculty who are interested in running project-based virtual internships. We outline some guidance and provide several resources to the reader as well as students’ observations and feedback. In addition to several Ten Rules articles on traditional internships, a few practical guides exist for faculty and employers who are interested in virtual internships [18,19].

It is our belief that virtual internships provide an excellent opportunity for faculty to support talent that is interested in computational projects, while such internships also create opportunities for growth for the supervisors in turn. The fact that virtual internships do not necessitate on-site resources and lab space makes these experiences an excellent complementary alternative to more location-bound internships. Given the right preparation, we believe that such internships may also carry collaborative potential outside of educational institutions. Indeed, virtual internships may enable faculty and students to collaborate with commercial entities on new projects. This is indeed already the case for many virtual interns who major in business-related or computer science fields.
